# “Perfect Leader, Perfect Leadership?” Linking Leaders’ Perfectionism to Monitoring, Transformational, and Servant Leadership Behavior

**DOI:** 10.3389/fpsyg.2021.657394

**Published:** 2021-04-16

**Authors:** Kathleen Otto, Hannah V. Geibel, Emily Kleszewski

**Affiliations:** Work and Organizational Psychology Unit, Institute of Psychology, Philipps University of Marburg, Marburg, Germany

**Keywords:** monitoring, servant leadership, multidimensional perfectionism, social disconnection, effective leadership

## Abstract

Despite the growing interest in perfectionism and its many facets, there is a lack of research on this phenomenon in the context of leadership. Attending to this deficit, the present study is the first to investigate the relationship between the three facets of perfectionism (self-oriented, socially prescribed, and other-oriented perfectionism) and three types of self-rated leadership behavior. In Study 1 (*N* = 182), leaders’ perfectionism and its association to their organizational, goal-oriented leadership behavior—self-rated as transactional (management by exception) and transformational leadership—is explored. In Study 2 (*N* = 185), the relationship of leaders’ perfectionism to their servant leadership as a people-centered leadership behavior is investigated. In line with the perfectionism social disconnection model (PSDM), we assume other-oriented and socially prescribed perfectionism to be positively related to management by exception (i.e., monitoring behavior) and negatively related to transformational and servant leadership, whereas the opposite pattern is primarily predicted for self-oriented perfectionism. Our findings in Study 1 reveal a negative relationship between leaders’ self-oriented perfectionism as well as positive relationships to their other-oriented and socially prescribed perfectionism in management by exception, while no substantial correlations with transformational leadership have emerged. In Study 2, a negative association between other-oriented perfectionism and the forgiveness dimension of servant leadership is revealed, indicating a possible barrier to building interpersonal relationships of acceptance and trust. Additionally, self-oriented perfectionism has been proven to be a rather favorable trait in servant leadership.

## Introduction

Setting ambitious goals and performing ideally is highly esteemed and desired, particularly in Western society; consequently, a certain degree of perfectionism is almost taken for granted (Spitzer, 2016) and substantially relates to engagement and motivation (Harari et al., 2018). However, Forbes’ (2018) article “Overcoming the leadership perfection problem” (Britcher, 2018) suggests that perfectionism may be a key problem for leadership. In practice, the topic is of significant concern; consulting firms offer coaching for leaders on managing the pitfalls of perfectionism, whereas in science (to the best of our knowledge) the impact of perfectionism on leadership has not yet been considered (Ocampo et al., 2020). While perfectionism has been widely researched, especially with regard to its negative implications in the clinical context (see Egan et al., 2011; Limburg et al., 2017, for a review and meta-analysis), the lack of research regarding its impact on leadership is unexpected, as this paucity was mentioned 15 years ago (Flett and Hewitt, 2006). With this study, we propose that leaders’ perfectionism is central to effective leadership behavior, and we explore how facets of perfectionism relate to specific forms of leadership behavior.

Effective leadership indicates that subordinates are satisfied and committed, and their business units are high performing (Luthans, 1988). As suggested by Hogan and Kaiser (2005), the leader’s personality determines the performed leadership behavior. Further, recent models by Derue et al. (2011) and Zaccaro et al. (2018) integrate the leader’s personality in a broader conceptual framework of leadership in which leader traits directly result in specific behaviors. De Vries (2012) empirically supports these suggestions by revealing strong relationships between personality and the majority of leadership behaviors. Early research on leadership has focused on leaders’ personal attributes, such as traits and skills. While skills are conceptualized as the ability to perform effectively—for example, interpersonally (Boyatzis, 1982)—traits are regarded as relatively stable dispositions to specific behavior (Yukl, 2009). For example, leadership motives and locus of control (McClelland and Boyatzis, 1982; Howell and Avolio, 1993) are related to leadership effectiveness. We build on these conceptual frameworks and consider personality traits as antecedents of leadership, and we propose that perfectionism is a relevant individual difference in leadership behavior.

Overall, this study contributes to the literature in two ways: We aim to extend knowledge in personality psychology regarding perfectionism in its application to the leadership context. *Perfectionism*—as a neglected personal attribute in leaders—is defined as a personality trait that is characterized by striving for flawlessness and having exceptionally high standards for performance combined with tendencies to evaluate one’s own or others’ behavior in an overly critical manner (Frost et al., 1990; Flett and Hewitt, 2002). Second, from the perspective of organizational psychology, we aim to broaden our understanding of effective leadership behavior by exploring a possible antecedent. Since perfectionism is seen as a multidimensional construct, the question arises regarding which dimensions of perfectionism may be detrimental or beneficial for effective leadership behavior.

## Theory Section

In the following, we first describe the tripartite concept of perfectionism along with the perfectionism social disconnection model (PSDM). Next, we introduce our relevant leadership concepts and aim to link leaders’ perfectionism with the way they lead.

### The Tripartite Model of Perfectionism

Early approaches describe perfectionism as a one-dimensional construct (e.g., Burns, 1980); however, the model that currently seems most appropriate is a multidimensional conceptualization that considers that psychological processes and consequences may differ between various dimensions of perfectionism (Stoeber, 2018a). The multidimensional model of perfectionism, introduced by Hewitt and Flett (1991), is one of the most common models in perfectionism research (Stoeber et al., 2018). Acknowledging that perfectionism has not only personal but also social aspects, the model and its associated measure—the multidimensional perfectionism scale (MPS; Hewitt and Flett, 1991, 2004)—propose three dimensions of perfectionism depending on source and direction: self-oriented, socially prescribed, and other-oriented perfectionism.

*Self-oriented perfectionism* represents the intrapersonal form of perfectionism and reflects unrealistic standards and expectations that are internally motivated and directed toward the self. It involves the assumption that striving for exceedingly high goals and achieving perfection are of particular importance. Additionally, self-oriented perfectionists are highly self-critical if they fail to meet their own standards. This dimension has been described as an ambivalent form of perfectionism associated with both positive and negative outcomes (Enns and Cox, 2002).

*Socially prescribed perfectionism* is an interpersonal form of perfectionism and includes beliefs that others have exceptionally high standards and expectations. Though directed toward the self, this form of perfectionism derives from the perception that others attach considerable importance to being perfect. Socially prescribed perfectionists fear negative social evaluations and express concern over obtaining others’ approval; consequently, this dimension is consistently related to psychological distress (Enns and Cox, 2002; Stoeber et al., 2021).

As opposed to the previous dimensions, *other-oriented perfectionism* is not focused on the self but on others. This dimension describes an important interpersonal form of perfectionism that involves extreme standards and expectations toward others. Other-oriented perfectionists believe that it is essential for others to be perfect, and they are highly critical of others, especially if the others fail to meet these expectations. Hewitt and Flett (1991) suggest that other-oriented perfectionism leads to behaviors such as blame, distrust, and hostility toward others. Unlike self-oriented and socially prescribed perfectionists, other-oriented perfectionists do not personally experience distress, but the targets of their demands do (Hewitt et al., 1995; Hewitt and Flett, 2004).

Interpersonal characteristics and behaviors are central in the PSDM (Hewitt et al., 2006), which represents an integrative theoretical framework that explains how perfectionism contributes to psychopathology through adverse social interactions, cognitions, and associated negative outcomes (Sherry et al., 2016). In brief, the model outlines social disconnection as a mechanism for the relationship between perfectionism and psychopathology. According to the model, perfectionism is related to unpleasant interpersonal characteristics and behaviors that lead to difficulties in developing and maintaining social relationships. The initial social disconnection model refers only to socially prescribed perfectionism and attributes key roles to interpersonal hostility and high interpersonal sensitivity (Hewitt et al., 2006). The expanded version of the model additionally applies to both of the other forms of perfectionism (Sherry et al., 2016; Hewitt et al., 2017). In other-oriented perfectionism, exceedingly high demands toward others that are accompanied by hostility, dominance, and disappointment may strain relationships (Sherry et al., 2007) and lead to impaired social contacts and feelings of isolation as others distance themselves (Hewitt et al., 2017).

Concerning self-oriented perfectionists, Sherry et al. (2016) argue that their strong ambitions are likely to result in a hostile and competitive mentality. Self-oriented perfectionists may favor competition over cooperation, which consequently leads to social disconnection. Socially prescribed perfectionism and other-oriented perfectionism consistently demonstrate positive relationships with indicators of social disconnection; however, this is not the case for self-oriented perfectionism (Stoeber et al., 2017). On the contrary, self-oriented perfectionism is positively related to empathy, trust, caring for others, and social goals in a recent series of studies, which indicates connection and a prosocial orientation (Stoeber, 2014; Stoeber et al., 2017).

The PSDM is a valuable framework that has been applied to investigate interpersonal consequences of perfectionism in several contexts, such as private conflicts (Mackinnon et al., 2012) or social exclusion and workplace conflicts (Kleszewski and Otto, 2020). In the present work, we aim to apply the core assumptions of the model concerning perfectionism, rigid cognitions, and adverse social interactions to the context of leadership behavior. We consider early conceptualizations of leadership as “a relation that exists between persons in a social situation” (Stogdill, 1974, pp. 63–64); therefore, we examine leadership as another social context in which perfectionism unfolds. Thus, we consider the PSDM to be especially relevant for leadership behavior that includes relational aspects and prosocial components (in our case, transformational leadership and servant leadership).

### Bridging Leaders’ Perfectionism With Their Leadership Behavior

Research on leadership can be categorized in diverse ways, with approaches that see leadership as an individual disposition, the behavior of an individual, or a social exchange process. Whereas early research investigated the individual dispositions of leaders, focus soon shifted to the behaviors performed by leaders and their behavioral constraints. More recently, leadership has been conceptualized as a social exchange process. However, the oldest leadership approach, i.e., exploring personal characteristics of the leader have regained attention in leadership research in recent years; for example, studies have examined the role of a proactive personality or the Big Five for leadership behavior (e.g., Lam et al., 2018; Sun and Shang, 2019). Building on this research, we argue that leaders’ perfectionism could be another personality antecedent that shapes effective leadership behavior. A recent study so far has linked leader perfectionism (not assessed a multidimensional concept though) with leader abusive behavior (Guo et al., 2020).

Notably, research on effective leadership behavior has been substantially influenced by the Ohio State and Michigan leadership studies from the 1950s, which reveal that subordinates view their leaders’ behavior primarily in terms of two categories: consideration and initiating structure (Katz et al., 1951; Katz and Kahn, 1952; Fleishman, 1953). We consider this early distinction in our research by selecting both kinds of leadership behaviors. Specifically, *consideration* relates to leaders demonstrating concern for their subordinates and for interpersonal relationships, while *initiating structure* refers to leader behavior that focuses on task accomplishment. Even today, 70 years later, recent leadership approaches can be classified in forms that are more task- or organizationally goal-oriented and those that are more people-centered. To reflect this differentiation, we explore leadership behavior that is described as monitoring or transformational as being primarily goal-oriented; servant leadership is considered here as being mainly people-centered.

In 1978, James MacGregor Burns first introduced the concept of transactional and transformational leadership (Burns, 1978), which can be classified as a goal-oriented way to lead. The model was expanded by Bass (1985b), and today the theory of transformational and transactional leadership is one of the most dominant concepts in leadership research (Judge and Piccolo, 2004); accordingly, it has attracted a substantial amount of research (Waldman et al., 1990; Tatoglu and Erkutlu, 2008; Hoch et al., 2018). Transactional leaders who motivate their subordinates through punishment and reward utilize the dimensions of management by exception (passive and active) for which negative associations with supervisor effectiveness, subordinates’ motivation, and performance and satisfaction with the leader have been noted (Judge and Piccolo, 2004; Hinkin and Schriesheim, 2008). In contrast, transformational leaders inspire their subordinates to perform better than expected, which relates to enhanced job satisfaction, team performance, and mental health (e.g., Braun et al., 2013; Scheel et al., 2019). Goal-oriented leadership behavior is reflected in the first study of the presented research through our exploration of perfectionism as an antecedent of transactional and transformational leadership behavior.

The demand for more ethical and considerate behavior in organizational contexts and outcomes, such as employee well-being and innovation (Van Dierendonck, 2011), has caused leadership research to shift its focus toward relational or people-centered ways of leadership (Avolio et al., 2009). Against this background, servant leadership has recently attracted researchers’ attention (see Van Dierendonck, 2011; Parris and Peachey, 2013; Eva et al., 2019; for reviews). Concerning outcomes, servant leadership is related to greater effectiveness on individual and organizational levels as well as enhanced well-being among subordinates (Parris and Peachey, 2013; Eva et al., 2019). According to a recent meta-analysis, this leadership style adds incremental variance beyond transformational leadership in outcomes such as employee engagement, work satisfaction, and commitment (Hoch et al., 2018). These results emphasize servant leadership as a promising research approach. Nevertheless, there is limited evidence that relates the characteristics of the leader to servant leadership behavior, and antecedents require further examination (Parris and Peachey, 2013; Liden et al., 2014; Eva et al., 2019). Hence, in our second study, we consider the dimension of people-oriented leadership and explore the relationship of leaders’ perfectionism to their servant leadership behavior. By doing so, we assume that both other-oriented and socially prescribed perfectionism in leaders strengthen less effective leadership behavior (management by exception), while leaders’ self-oriented perfectionism relates to effective leadership behavior (transformational and servant leadership) and vice versa.

## Empirical Studies

In the following, we describe the two empirical studies conducted with German leaders who answered questions regarding how perfectionism shapes their leadership behavior. Study 1 focuses on the relationship of leaders’ perfectionism to goal-oriented leadership behavior (i.e., transactional and transformational leadership), whereas Study 2 aims to elucidate the people-centered approach of servant leadership. For each study, we first introduce the respective leadership theory and then derive the hypotheses by linking the theory to potential antecedents in the facets of other-oriented, socially prescribed, and self-oriented perfectionism.

### Study 1: Perfectionism and Goal-Oriented Leadership

Study 1 serves three goals: Initially, we aim to provide evidence that there is indeed a relationship between perfectionism in leaders and their leadership behavior (proof of concept). Second, by applying two opposing goal-oriented leadership approaches—transactional and transformational leadership—we underscore that the association of leaders’ perfectionism and leadership behavior is true for more than one type of behavior. Third, by considering perfectionism on its facet level, we intend to detect a differentiated pattern of more adaptive and maladaptive links between leaders’ perfectionism and their method of leadership.

#### Transactional and Transformational Leadership

Transactional leadership behavior is characterized as a social exchange process in which subordinates are made aware of what they are expected to do to receive rewards or avoid punishment (Bass, 1985a, b, 1990; Yukl, 1999; van Eeden, 2008). To guarantee this, transactional leaders exhibit three types of behavior that motivate subordinates by appealing to their own self-interest—contingent reward as well as passive and active management by exception (Bass, 1985a, 1990). In our study, we focus on management by exception, as this type of leader behavior may be based on perfectionism because it regards maintaining standards. The passive type of management by exception describes a leader who reacts to mistakes, errors, and problems when they occur. A leader who demonstrates active management actively monitors the performance of subordinates and looks forcefully for mistakes, irregularities, exceptions, deviations from standards, and failures in an attempt to correct them before they occur (van Eeden, 2008; Finckler, 2017).

In Bass’ model, transformational leadership behavior does not react to circumstances; rather, the leader performing transformational leadership inspires subordinates and influences their beliefs, needs, and values (Burns, 1978; Kuhnert and Lewis, 1987). Hence, transformational leadership aims to enhance the intrinsic motivation by offering people a higher sense of purpose in their work (Bass and Riggio, 2006). According to Bass and Avolio (1995), transformational leadership consists of four components: *Idealized influence* means that leaders conform to ethical standards and prioritize team interests over their personal interests. This behavior stimulates subordinates’ respect and trust in the leader. *Inspirational motivation* refers to the enthusiasm and optimism of the leader who creates a vision of the future for subordinates. High performance expectations, specific goals, and commitment to the vision are clearly communicated. *Intellectual stimulation* is described as the behavior of valuing and challenging subordinates’ intellectual abilities to promote creativity and innovation. Finally, *individual consideration* describes the leader’s attempt to assume subordinates’ perspectives and recognize their individual needs. The leader acts as a mentor and provides advice, feedback, and support (Bass, 1985a; Eisenbeiss et al., 2008; van Eeden, 2008).

#### Leaders’ Perfectionism and Monitoring Versus Transformational Leadership

As described, the three dimensions of perfectionism are characterized by different motivations and behaviors, which suggests that self-oriented perfectionism, other-oriented perfectionism, and socially prescribed perfectionism are differently associated with transactional (in this case, management by exceptions, both active and passive) and transformational leadership behavior.

Overall, perfectionists are known for being critical, which should also be linked to monitoring of provided performances (Stoeber, 2018a). Leadership behavior that is related to fulfilling organizational goals and is described by the facet of management by exception contains monitoring and controlling processes and outcomes (van Eeden, 2008; Finckler, 2017). Yet, as self-oriented perfectionists focus on their own goals and not those of others, a specific assumption regarding self-oriented perfectionism cannot be developed.

Socially prescribed perfectionism is described as the key indicator of perfectionistic concerns—which may explain why socially prescribed perfectionists are actively hunting mistakes and discrepancies (Stoeber, 2018a). This leads to the assumption that socially prescribed perfectionists should demonstrate a higher active management by exception in their leadership behavior. Other-oriented perfectionists should additionally perform active management by exception, assuming that the criticism is concentrated on the performance and standards of others. Hence, the two dimensions of perfectionism with a social focus should be positively linked with active management by exception. Following this line of thinking, it is logical to assume that other-oriented and socially prescribed perfectionists would also indicate a positive association with passive management by exception, as leaders with such personalities would react to any mistakes that may occur.

H1a: Other-oriented perfectionism is positively related to management by exception.
H1b: Socially prescribed perfectionism is positively related to management by exception.

Furthermore, in transformational leadership, the leaders’ perfectionism may explain the occurrence of this positive form of goal-oriented leadership behavior. In line with the PSDM, both other-oriented perfectionism and socially prescribed perfectionism are negatively associated with a prosocial orientation and positively associated with uncaring traits, both of which indicate antisocial facets (Stoeber, 2018a). Additionally, other-oriented perfectionism and socially prescribed perfectionism are found to be inversely related to agreeableness (Stoeber, 2014; Smith et al., 2019). These prior findings speak against the idea of leading in a transformational way and especially against the ideas of addressing followers’ needs and assuming their perspectives. The PSDM proposes that other-oriented and socially prescribed perfectionists have difficulties developing and maintaining social relationships, whereas transformational leaders demonstrate a behavior of intellectually stimulating their subordinates and caring about their worries and concerns to enable fulfillment of organizational goals. Moreover, socially prescribed perfectionism’s focus on avoiding mistakes could cause higher-level, long-term organizational goals to become out of focus. Hence, these two dimensions of perfectionism should be negatively linked with transformational leadership behavior.

Self-oriented perfectionists, however, are described as being conscientious, behaving altruistically and prosocially, and indicating interest in others (Stoeber, 2014; Smith et al., 2019). While focusing primarily on organizational goals and providing a vision, transformational leadership still considers each individual on the team in this way, for which a prosocial attitude is necessary. Transformational leadership is predicted not only by extraversion, but also by conscientiousness (Bono and Judge, 2004); therefore, self-oriented perfectionism may have a positive correlation with transformational leadership behavior.

H2a: Other-oriented perfectionism is negatively related to transformational leadership.
H2b: Socially prescribed perfectionism is negatively related to transformational leadership.
H2c: Self-oriented perfectionism is positively related to transformational leadership.

#### Method

##### Sampling criteria and procedure

Data were collected through an online survey posted on social media platforms (such as XING and Facebook), job search forums, and mailing lists in Germany in 2017. As a preselection criterion, potential participants should be in a leadership position with responsibility for at least one subordinate. Participation was voluntary, and no compensation was offered.

Overall, 216 people completed the survey. The data were checked for potential dropouts, and 25 cases were excluded, as they did not meet the requirements of the study. Another eight participants were eliminated from the dataset because their total retention period was less than 200 s, which indicated that the survey was not completed with enough attention. Further, one case illustrated a Mahalanobis distance larger than the critical value of χ^2^(6) = 22.46, *p* < 0.001 (Tabachnick and Fidell, 2007) and was therefore excluded.

##### Sample description

The final sample included 182 leaders. The ages ranged from 19 to 63 (*M* = 38, SD = 10.69); among the participants, 109 were female, and 72 were male (one person did not indicate their gender). They primarily worked in public service (60.4%), while 29.7% were employed in the private sector. The majority were working for a company with more than 500 employees (39.7%) or 100–500 employees (39.7%); 17.9% responded that they were working in a company with 31–100 employees, 12% with 11–30 employees, and 13% with less than 10 employees. Nearly one-quarter (24.7%) had an organizational tenure of more than 10 years, and the majority of leaders had spent between 6 months and 2 years or between 2 and 5 years (each 30%) in their current position.

With 46 participants, the major part of the sample was responsible for three to five subordinates. Moreover, 31 stated that they were responsible for six to 10 employees, another 31 participants had 11–20 subordinates, 24 were in charge of two subordinates, and 33 were responsible for more than 20 subordinates. Regarding the frequency of contact between leaders and subordinates, more than one-half of the leaders (59.9%) stated that they had contact every day, while 27.5% said that their contact was several times a week. Fewer participants had less contact, such as once a week (5.5%), several times a month (6.6%), or less than once a month (0.5%).

##### Measures

*Perfectionism*. To assess perfectionism, a 15-item short form of the Multidimensional Perfectionism Scale (HF-MPS; Hewitt and Flett, 1991; German translation: Altstötter-Gleich, 1998) was utilized to measure self-oriented perfectionism (five items; e.g., “One of my goals is to be perfect in everything I do,” α = 0.84), socially prescribed perfectionism (five items; e.g., “People expect nothing less than perfection from me,” α = 0.86), and other-oriented perfectionism (five items; e.g., “Everything that others do must be of top-notch quality,” α = 0.71). Following Stoeber (2018b), self-oriented and socially prescribed perfectionism were measured with the short form proposed by Cox et al. (2002), whereas other-oriented perfectionism was assessed through the short form published by Hewitt et al. (2008). Items were presented with the MPS’ standard instruction (“Listed below are a number of statements concerning personal characteristics and traits…”) and were answered on a 7-point Likert scale ranging from “disagree” to “agree.” Cronbach’s alpha of the total scale was α = 0.88.

*Monitoring leadership behavior*. We assessed management by exception—as a transactional leadership behavior—with the German translation (Felfe, 2006) of the Multifactor Leadership Questionnaire (MLQ; Bass and Avolio, 1995). The passive and the active subscale contained four items each which were rated on a 5-point Likert scale ranging from “rarely or never” to “very frequently, if not always.” Active management by exception (e.g., “I focus my attention on irregularities, mistakes, exceptions, and deviations from what I expect”) yielded a Cronbach’s alpha of 0.64 and thus failed the criterion of alpha >0.70, which is required to be considered satisfactory (Nunnally and Bernstein, 1994). Alpha is dependent on the length and breadth of a measure; however, it is also important to consider inter-item correlations, particularly for short scales (Streiner, 2003). The mean inter-item correlation was acceptable with an *r*_est_ of 0.30, which corresponds to a Cronbach’s alpha of 0.80 for a 10-item long measure. For passive management by exception (e.g., “Problems have to be chronic before I take action”), the Cronbach’s alpha was detected to be acceptable after excluding one item (α = 0.61, *r*_est_ = 0.36).

*Transformational leadership behavior*. Transformational leadership has often been criticized for a lack of conceptual distinctiveness (van Knippenberg and Sitkin, 2013), and empirical research has failed to reproduce the four-dimensional structure proposed by Bass and Avolio (1995); therefore, we assessed transformational leadership as a one-dimensional construct, utilizing the global transformational leadership scale by Carless et al. (2000) as a self-appraisal. The short scale contains seven items (e.g., “I treat staff as individuals, support and encourage their development”); however, it indicated high convergent validity with other measurements, such as the MLQ (Carless et al., 2000). The seven questions were presented with the same answering mode that we applied to the transactional leadership behavior. The reliability of the scale within this study illustrated an acceptable Cronbach’s alpha with α = 0.77.

##### Results and Discussion

As presented in Table 1 and in accordance with previous findings (e.g., Hewitt and Flett, 2004; Stoeber and Otto, 2006; Stoeber, 2014), other-oriented, socially prescribed, and self-oriented perfectionism are significantly positively correlated, which indicates a substantial overlap.

**TABLE 1:** Descriptive statistics, reliabilities, and intercorrelations of the measures in study 1.

	**1**	**2**	**3**	**4**	**5**	**6**
**Multidimensional Perfectionism**						
1. Other-oriented perfectionism	(0.74)					
2. Socially prescribed perfectionism	0.26**	(0.86)				
3. Self-oriented perfectionism	0.42**	0.23**	(0.85)			
**Leadership Behavior**						
4. Management by exception – Active	0.30**	0.25**	0.24**	(0.64)		
5. Management by exception – Passive	0.07	0.28**	−0.07	−0.00	(0.60)	
6. Transformational leadership	0.03	−0.08	0.09	0.69	−0.13	(0.76)
*M*	4.40	2.88	5.42	2.89	2.13	4.13
SD	1.02	1.30	0.98	0.63	0.65	0.47

*N* = 182 leaders. Correlations according to Pearson. Cronbach’s alpha in brackets.

*p < 0.05 and **p < 0.01.

Our hypotheses suggested that other-oriented (H1a) and socially prescribed perfectionism (H1b) would be positively linked with both forms of management by exception. Additionally, we assumed that other-oriented (H2a) and socially prescribed perfectionism (H2b) should be negatively correlated with transformational leadership behavior, while self-oriented perfectionism (H2c) should indicate a positive correlation with this positive goal-oriented leadership behavior.

Stoeber and Gaudreau (2017) state that the investigation of perfectionism requires multivariate analyses, as unique relationships between the specific dimensions may vary due to the multidimensional nature of the construct. Hence, hierarchical regression analyses were utilized to test the hypotheses and to simultaneously account for the three perfectionism dimensions. As gender and age are important demographic variables to consider in leadership research (e.g., Barbuto et al., 2007), they were considered to be control variables in the first step of the regression. In the second step, the three perfectionism dimensions were added. The results are displayed in the left part of Table 2.

**TABLE 2:** Explaining leadership behavior by demographic variables and multidimensional perfectionism.

		**Study 1**			**Study 2**								
**Step**	**Variable**	**Mbe-a**	**Mbe-p**	**TL**	**SL**	**Emp**	**Sta**	**Acc**	**Forg**	**Cour**	**Aut**	**Hum**	**Stew**
**Step 1**													
	Gender (1 = male)	0.12	0.17*	−0.10	−0.08	−0.19*	0.06	−0.13	−0.06	0.23**	−0.25**	−0.12	−0.01
	Age	−0.08	−0.16*	0.07	0.20*	0.20*	0.08	0.13	0.07	0.11	0.05	0.22**	0.04
	Δ *R*^2^	0.02	0.05*	0.01	0.04*	0.06**	0.01	0.04*	0.00	0.05**	0.05**	0.05*	0.00
**Step 2**													
	Other-oriented perfectionism	0.19*	0.02	0.03	−0.03	0.06	−0.04	0.17^†^	−0.36**	0.06	−0.07	0.04	0.03
	Socially prescribed perfectionism	0.18*	0.30**	−0.11	−0.04	−0.03	0.00	0.07	−0.15^†^	−0.08	0.12	−0.10	0.01
	Self-oriented perfectionism	0.12	−0.16*	0.11	0.16^†^	0.01	0.03	0.07	0.03	0.15^†^	−0.03	0.18*	0.19*
	Δ*R*^2^	0.13**	0.10**	0.02	0.02	0.00	0.00	0.06**	0.19**	0.03	0.01	0.03	0.04
	*F*	5.83**	5.75**	1.20	2.17	2.24	0.51	4.12**	8.41**	3.02*	2.29*	3.09*	1.63

*Study 1: *N* = 182 leaders. Study 2: *N* = 185 leaders. Mbe-a, management by exception active; Mbe-p, management by exception passive; TL, transformational leadership; SL, servant leadership (composite score); Emp, empowerment; Sta, standing back; Acc, accountability; Forg, forgiveness; Aut, authenticity; Hum, humility; Stew, stewardship.*

^†^*p* < 0.10, *p < 0.05, and **p < 0.01.

Other-oriented perfectionism as well as socially prescribed perfectionism was positively related to active management by exception, thus providing support for H1a and H1b. The higher the leaders’ other-oriented and socially prescribed perfectionism, the more they reported monitoring leadership behavior, such as screening the work of their subordinates for any mistakes, irregularities, or deviations from standards. Passive management by exception, in contrast, was predicted by socially prescribed perfectionism and self-oriented perfectionism. As assumed in H1b, socially prescribed perfectionistic leaders illustrated more passive management by exception, such as immediately reacting to errors or problems. Moreover, younger leaders as well as male leaders employed more passive management by exception. No substantial relationships to other-oriented perfectionism were found, thus contradicting H1a.

Moreover, self-oriented perfectionistic leaders reported less passive management by exception and therefore less correctional behaviors. Though no hypothesis referring to this link was theoretically derived, self-oriented perfectionism in leaders demonstrated adaptive consequences for subordinates. Self-oriented perfectionistic leaders seem to guarantee subordinates higher autonomy, which may enable a more constructive error culture in an organization.

Finally, none of the perfectionism dimensions reveal any significant relationships to transformational leadership. Hence, hypotheses H2a–c must be rejected. Several *post hoc* explanations may indicate why we failed to find the proposed relationships between perfectionism and transformational leadership: We decided to measure transformational leadership as a one-dimensional construct (Carless et al., 2000) because the four-dimensional construct—comprising the facets of idealized influence, inspirational motivation, intellectual stimulation, and individual consideration—is empirically difficult to prove (van Knippenberg and Sitkin, 2013). However, such a global measure may have masked the true correlations between the multidimensional construct of perfectionism and the multidimensional construct of transformational leadership.

It seems to be plausible that when examining the facet level, relationships between multidimensional perfectionism and transformational leadership would have appeared. In particular, the dimension of individual consideration, which contains the prosocial orientation necessary to focus individually on followers, may have been positively linked to self-oriented perfectionism and negatively to other-oriented and socially prescribed perfectionism. Moreover, in a similar vein, one could argue that for the dimension of idealized influence, which refers to the fact that leaders prioritize team interests over their personal interests (Bass and Avolio, 1995), a prosocial orientation enters, which, in accordance with the PSDM, should be based on lower other-oriented and lower socially prescribed perfectionism. Such links are more difficult to theoretically develop for the other dimensions, such as inspirational motivation and intellectual stimulation—which may be the reason that we did not find significant relationships on the global level.

However, transformational leadership—independent of whether it is assessed multidimensionally or as a broad, one-dimensional concept—may remain an excessively goal-oriented way to lead. Regarding the predicted positive impact of self-oriented perfectionism, leadership behavior that primarily focuses on people (not tasks or goals) should be examined.

#### Study 2: Perfectionism and People-Centered Leadership

Study 2 investigates a different leadership concept that does not primarily focus on the goals of an organization, as transactional (or monitoring) and transformational leadership behaviors do. While both transformational and servant leadership share the idea of motivating or empowering subordinates, servant leadership has been distinguished from transformational leadership by its main focus, which is directed not toward the organization but primarily toward the individuals (Stone et al., 2004). Servant leadership represents a people-centered leadership behavior that emphasizes the needs and interests of the subordinates (Van Dierendonck, 2011; Eva et al., 2019). In their study, Parolini et al. (2009) confirm this assumption, revealing that transformational leaders were perceived as focusing on organizational goals, while servant leaders were seen as prioritizing the needs of the individuals. The idea that unpleasant interpersonal behavior and the resulting social disconnection are inherent in interpersonal forms of perfectionism may be particularly problematic for demonstrating people-centered leadership behavior. Hence, the second study adds knowledge regarding the role of leaders’ perfectionism in a particularly follower-related approach.

##### Defining Servant Leadership Behavior

With the publication of his essay “The Servant as Leader” in 1970, Greenleaf introduced the fundamental ideas of servant leadership (Greenleaf, 1970). Overall, it can be described as a people-centered and more ethical (Clegg et al., 2007) leadership theory (Van Dierendonck, 2011). A special attribute of this theory is that leaders act beyond self-interest by focusing on the needs of their subordinates (Van Dierendonck, 2011). Additionally, servant leadership has been named “the only leadership approach that frames the leadership process around the principle of caring for others” (Northouse, 2016, p. 240). In a recent review, Van Dierendonck (2011) has identified a number of key characteristics that can be viewed as an operationalized definition of the construct. Based on these characteristics, Van Dierendonck and Nuijten (2011) have developed the Servant Leadership Survey (SLS). As their approach comprises an integration of previous servant leadership theories and research (Pircher Verdorfer and Peus, 2014), the present study adapts their conceptualization.

According to Van Dierendonck and Nuijten (2011), servant leadership consists of the following eight dimensions:

*Empowerment* involves behavioral aspects, such as sharing information, delegating tasks, encouraging self-directed decision making, and developing skills (Konczak et al., 2000) and is intended to create a feeling of individual power (Van Dierendonck, 2011). By empowering their subordinates, servant leaders acknowledge the intrinsic value of each person and their potential to learn and develop (Greenleaf, 1998).

The dimension of *standing back* refers to the degree to which leaders focus on the needs of others instead of prioritizing their own interests. Consequently, servant leaders also stay in the background when success is attained (Van Dierendonck and Nuijten, 2011). The third dimension, *accountability*, describes the fact that individuals and teams are considered responsible for outcomes (Konczak et al., 2000). Therefore, leaders provide a framework for their subordinates’ autonomy and exhibit confidence in their capabilities (Van Dierendonck and Nuijten, 2011). *Forgiveness* aims to form interpersonal relationships of high quality by avoiding resentment and hostility if mistakes occur (Van Dierendonck and Nuijten, 2011). Servant leaders intend to create an environment of interpersonal acceptance and trust (Ferch, 2005).

*Courage* is characterized by taking chances and by responding to problems with new approaches (Greenleaf, 1977). According to Van Dierendonck and Nuijten (2011), courage includes behaving in a proactive way and is relevant in creativity and innovation. Further, servant leadership comprises *authenticity*. This dimension emphasizes that leaders act in consistence with their feelings and inner thoughts, remaining true to themselves (Harter, 2002).

*Humility* relates to leaders’ awareness of their strengths and weaknesses as well as their openness to overcome these weaknesses with the support of others (Morris et al., 2005). Thus, servant leaders acknowledge that they are fallible themselves.

As described by the authors, the last dimension, *stewardship*, is linked to the idea of responsibility for the common good. Extending beyond self-interest, leaders should not merely care for their subordinates, but they should represent a role model who encourages others to act in their mutual interest (Hernandez, 2008).

##### Leaders’ Perfectionism and Servant Leadership Behavior

Leaders’ type and level of perfectionism is relevant to whether they engage in servant leadership. It can be argued that other-oriented perfectionism and socially prescribed perfectionism represent barriers to servant leadership. The prioritization of the subordinates’ needs requires a certain level of social orientation. As captured in the PSDM, both interpersonal dimensions of perfectionism describe difficulties in developing and maintaining social relationships. Consistent with the PSDM, other-oriented perfectionism and socially prescribed perfectionism portray negative relationships with agreeableness (Smith et al., 2019). Agreeableness, however, was found to be an antecedent of servant leadership on a personality level (Hunter et al., 2013). More specifically, other-oriented perfectionists impose excessive standards and expectations and are highly critical and hostile, which contrasts with the principle of caring for team members. As Greenleaf (1977) states, servant leadership involves an acceptance of imperfection because mistakes must be regarded as a part of human nature. Socially prescribed perfectionists are characterized by low altruism, high mistrust, and interpersonal hostility (Stoeber, 2014; Stoeber et al., 2017). These interpersonal characteristics and behaviors should make it difficult for socially prescribed perfectionistic leaders to prioritize their subordinates’ needs and display a follower-related leadership approach. Accordingly, we expect that other-oriented perfectionism and socially prescribed perfectionism are negatively related to servant leadership.

As described previously and contrary to the PSDM, self-oriented perfectionism presents a different pattern of relationships. Self-oriented perfectionists are characterized by social connection and a rather prosocial orientation, demonstrating empathy, trust, and care for the people around them (Stoeber, 2014; Stoeber et al., 2017). Accordingly, we expect an adaptive relationship between self-oriented perfectionism and the most people- or follower-centered leadership behavior: servant leadership.

H3a: Other-oriented perfectionism is negatively related to servant leadership.
H3b: Socially prescribed perfectionism is negatively related to servant leadership.
H3c: Self-oriented perfectionism is positively related to servant leadership.

It can be assumed that the relationships between perfectionism and servant leadership do not equally apply to all dimensions of servant leadership. Therefore, the strengths of the relationships between perfectionism and the specific dimensions are investigated on an exploratory basis. This approach provides specific insight into how perfectionism among leaders becomes apparent on the behavioral level.

##### Method

###### Sampling criteria and procedure

As in Study 1, the investigated sample consisted of people currently holding a leadership position with responsibility for at least one team member. In line with the idea of servant leadership behavior, we additionally recruited leaders from non-profit contexts as participants. Again, data were collected in an online survey that was posted on social media platforms (such as XING and Facebook), job search forums, and mailing lists in Germany in 2018. Participation was voluntary. As an incentive, each participant had the opportunity to participate in a lottery for two gift cards.

Overall, 238 participants completed the survey. Three participants were eliminated from the dataset, as they did not hold a leadership position. Moreover, as some items of the servant leadership measure refer to leaders’ behavior toward supervisors, another 42 participants were excluded because they had no supervisors, which would have caused missing data on these items and identification problems while conducting the confirmatory factor analysis (CFA). An additional eight participants were excluded from the analyses because they completed the survey in less than 200 s, which indicates a lack of attention during completion. As in Study 1, we checked the data for multivariate outliers utilizing Mahalanobis distance, but there was no value exceeding the critical value of χ^2^(11) = 31.26, *p* < 0.001 (Tabachnick and Fidell, 2007).

###### Sample description

A final sample of 185 participants was included in the analyses. The sample consisted of 126 women (68.1%) and 59 men (31.9%), with ages ranging from 18 to 65 years (*M* = 38.16, SD = 11.01). The sample was composed of leaders from all branches of the economy, among which human health and social work activities (16.8%), education (12.4%), information and communication (11.9%), manufacturing (8.1%), and public administration (8.1%) were the most frequent categories. The majority of the leaders were working in the private sector (51.4%), followed by the public sector (37.3%) and volunteer sector (a non-profit context; 11.4%). Concerning the managerial level, 76.8% of the participants indicated that they worked in lower management, 17.3% in middle management, and 5.9% in upper management. The mean work experience in a leadership position was 7.84 years (SD = 7.55), with an overall team tenure of 4.77 years (SD = 5.33). On average, leaders were responsible for 18.82 team members (SD = 33.19) and spent 19.82 h with them per week (SD = 13.89).

###### Measures

*Perfectionism*. Perfectionism was assessed in the same way as in Study 1, with all three facets revealing satisfying reliabilities: self-oriented perfectionism (α = 0.86), socially prescribed perfectionism (α = 0.87), and other-oriented perfectionism (α = 0.76). Scale scores were calculated by averaging responses across items.

*Servant leadership*. The German version (Pircher Verdorfer and Peus, 2014) of the SLS (Van Dierendonck and Nuijten, 2011) was employed to measure servant leadership. To capture thoughts on servant leadership from the leaders’ perspective, the original items were rephrased and converted into a self-rating version. The leaders were asked to answer the 30 items on a 6-point Likert scale ranging from 1 (“strongly disagree”) to 6 (“strongly agree”). The SLS encompasses eight subscales: Empowerment (seven items; e.g., “I help my team members to further develop themselves,” α = 0.81), standing back (three items; e.g., “I keep myself in the background and give credit to others,” α = 0.65), accountability (three items; e.g., “I hold my team members responsible for the work they carry out,” α = 0.73), forgiveness (three items; e.g., “I find it difficult to forget things that went wrong in the past,” α = 0.62), courage (two items; e.g., “I take risks and do what needs to be done in my view,” α = 0.83), authenticity (four items; e.g., “I show my true feelings to my team members,” α = 0.72), humility (five items; e.g., “I learn from criticism,” α = 0.83), and stewardship (three items; e.g., “I emphasize the importance of focusing on the good of the whole,” α = 0.60). Three of the subscales (standing back, forgiveness, and stewardship) failed to reach an internal consistency of 0.70 or above, which is required to be considered satisfactory (Nunnally and Bernstein, 1994). This finding is comparable to the reliabilities reported for the original measure, in which the standing back and stewardship dimensions also earned alphas below 0.70 (Pircher Verdorfer and Peus, 2014). It must be considered, however, that alpha is dependent on the length of a measure and that inter-item correlations should be examined (Streiner, 2003); these analyses yielded satisfactory values of between *r*_est_ = 0.33 and 0.38. A composite servant leadership score was constructed by averaging scale means. Cronbach’s alpha of the total scale was α = 0.82.

A CFA was conducted to investigate the factorial structure of the adapted self-rating version of the German SLS (Van Dierendonck and Nuijten, 2011; Pircher Verdorfer and Peus, 2014) utilizing Mplus (Version 6). As it was intended to replicate the postulated eight-dimensional structure, a multidimensional model in which the eight factors were allowed to correlate was tested. The eight-factor model fit the empirical data with a chi-square of 561.78 (*p* < 0.001), *df* = 377, CFI = 0.89, RMSEA = 0.05 [90% CI (0.04, 0.06)], SRMR = 0.07. A value lower than 0.90 for the CFI indicates a need to revise the model (Brown, 2006). Modification indices referring to error covariances were examined to detect possible misspecifications. Three main areas of misspecification were identified, which concerned the pairing of error terms associated with two items from the empowerment subscale, two items from the authenticity subscale, and two items from the humility subscale, which in the German version were characterized by similar content and wording. After the adaptations were implemented, the modified eight-factor model containing the three error covariances was determined to have an acceptable fit with the empirical data {χ^2^ = 492.41, *df* = 374, p < 0.001; CFI = 0.93; RMSEA = 0.04 [90% CI (0.03, 0.05)], SRMR = 0.06; Kline, 2005}. Accordingly, the postulated eight-dimensional model of the SLS was confirmed with fit indices close to the original versions (see Van Dierendonck and Nuijten, 2011; Pircher Verdorfer and Peus, 2014;, for comparison), which indicates factorial validity of the adapted self-rating version.

#### Results and Discussion

Descriptive statistics, reliabilities, and intercorrelations of the scales are presented in Table 3. As was true for leaders in Study 1, other-oriented, socially prescribed, and self-oriented perfectionism were positively related to each other, which indicates a need to control for the overlap.

**TABLE 3:** Descriptive statistics, reliabilities, and intercorrelations of the measures in study 2.

	**1**	**2**	**3**	**4**	**5**	**6**	**7**	**8**	**9**	**10**	**11**	**12**
**Multidimensional perfectionism**												
1. Other-oriented perfectionism	(0.76)											
2. Socially prescribed perfectionism	0.53**	(0.87)										
3. Self-oriented perfectionism	0.49**	0.32**	(0.86)									
**Servant leadership**												
4. Composite score	0.03	0.01	0.13	(0.82)								
5. Empowerment	0.08	0.02	0.06	0.64**	(0.81)							
6. Standing back	−0.04	−0.01	−0.01	0.60**	0.32**	(0.65)						
7. Accountability	0.27**	0.19**	0.20**	0.34**	0.18*	0.09	(0.73)					
8. Forgiveness	−0.41**	−0.32**	−0.18*	0.28**	0.12	0.14	−0.23**	(0.62)				
9. Courage	0.03	0.00	0.09	0.51**	0.16*	0.15*	0.09	−0.03	(0.83)			
10. Authenticity	0.04	0.08	0.04	0.51**	0.23**	0.17*	0.07	−0.01	0.09	(0.72)		
11. Humility	0.08	−0.01	0.17*	0.59**	0.46**	0.17*	0.16*	0.20**	0.11	0.29**	(0.83)	
12. Stewardship	0.12	0.01	0.20**	0.63**	0.44**	0.29**	0.03	0.00	0.25**	0.27**	0.32**	(0.60)
*M*	4.33	3.08	5.33	4.53	5.24	3.89	4.54	4.69	4.18	4.15	5.10	4.44
SD	1.07	1.38	1.05	0.45	0.58	1.06	0.93	0.92	1.14	0.95	0.65	0.94

*N* = 185 leaders. Correlations according to Pearson. Cronbach’s alpha in brackets. *p < 0.05 and **p < 0.01.

We expected other-oriented (H3a) and socially prescribed perfectionism (H3b) to negatively link to servant leadership, whereas we expected that self-oriented perfectionism (H3c) would be positively linked to this leadership behavior. The results of the regression analyses can be found in the right part of Table 2.

To explain the global concept of servant leadership using the composite score, in addition to the effect that older leaders employed more servant leadership behaviors, we found a marginally significant positive relationship of self-oriented perfectionism with the criterion, thus providing initial support for H3c. Self-oriented perfectionistic leaders reported performing more servant leadership behaviors.

In contrast, neither other-oriented nor socially prescribed perfectionism were associated with servant leadership on the global level, thus opposing H3a and H3b. Furthermore, the proposed negative relationships could only be confirmed on the facet level, in which other-oriented and marginally socially prescribed perfectionistic leaders revealed less forgiveness toward their followers. Specifically, such leaders experienced more problems when forming interpersonal relationships of high quality with their followers because they were resentful and behaved with hostility when mistakes occurred. Further, other-oriented perfectionism was marginally positively correlated with accountability, which implies that leaders who score high in other-oriented perfectionism consider their team members to be more responsible for outcomes than leaders who score low in this dimension. In light of the negative relationship with forgiveness, this positive association may reflect that other-oriented perfectionists consider other people to be responsible not only for desired outcomes, but also for their mistakes.

On the facet level, self-oriented perfectionistic leaders scored marginally higher in the dimensions of courage, humility, and stewardship, thus supporting the assumptions of H3c. Hence, the overall marginally positive relationship of self-oriented perfectionism to servant leadership behavior is based on the leaders’ ability to proactively take chances and respond to challenges with innovative approaches (which implies courage, which was demonstrated to be higher in male leaders in this sample), the leaders’ awareness of their strengths and weaknesses and their readiness to refine themselves (this implies humility, which was demonstrated to be higher in older leaders in this sample), and the leaders’ sense of responsibility for the mutual interest and common good (which involves stewardship).

Generally, the results provide further evidence for the relevance of the leader’s perfectionism to the performed leadership behavior. Additionally, the study leads to the conclusion that other-oriented perfectionism can be regarded as a barrier to building trusting relationships due to its negative relationship with the forgiveness dimension of servant leadership. Furthermore, self-oriented perfectionism is revealed to be a rather promotive trait concerning this leadership behavior. To summarize, leaders who score low overall in other-oriented perfectionism and high in self-oriented perfectionism may be favorable with regard to servant leadership.

## General Discussion

With this research, we have aimed to elucidate the under-researched topic of leaders’ perfectionism and the selection of their leadership behavior. The present study answers recent calls to explore how facets of perfectionism in supervisors impact their leadership behavior (Ocampo et al., 2020). Utilizing the three dimensions of perfectionism by Hewitt and Flett (1991) allowed us to examine a broad picture of this personality trait, illuminating that the different types of perfectionism reveal different relationships with the examined leadership behaviors. Guided by the PSDM, we assumed other-oriented and socially prescribed perfectionism to be positively related to management by exception (i.e., monitoring behavior) and negatively related to transformational and servant leadership, while we believed that the opposite pattern should be true for self-oriented perfectionism.

We could not provide evidence for a link between leaders’ perfectionism and transformational leadership; however, for monitoring (i.e., management by exception) and servant leadership behaviors, our findings support our assumptions. Self-oriented perfectionism has been linked to nurturance goals and altruism, indicating that self-oriented perfectionists are inclined to behave in a prosocial manner (Stoeber, 2014; Stoeber et al., 2018). Accordingly, self-oriented perfectionistic leaders were less likely to display management by exception (Study 1) and were more likely to manifest servant leadership behavior (Study 2). Our findings suggest some positive aspects of the self-oriented perfectionism facet for subordinates, as leaders who score high in self-oriented perfectionism focus on their own mistakes (as demonstrated by its relation to the humility facet of servant leadership) and do not seem to search for errors and mistakes made by their subordinates (less monitoring behavior). However, it remains unclear whether this indicates that subordinates of self-oriented perfectionists have the freedom and autonomy to develop themselves and their careers.

In contrast to these findings, other-oriented and socially prescribed perfectionism in leaders positively predicts management by exception (Study 1), and other-oriented perfectionistic leaders further demonstrate a lower tendency to forgive in case of mistakes (Study 2). Other-oriented perfectionism is one aspect of multidimensional perfectionism (Hewitt and Flett, 1991) that has received comparatively little research attention to date (Stoeber, 2014). However, the present study provides evidence that, in particular, this dimension may be detrimental in leadership. In line with this, other-oriented perfectionism has previously been described as the “dark” form of perfectionism, thus indicating positive correlations with the Dark Triad of personality (Stoeber, 2014; Marcus and Zeigler-Hill, 2015). Our results extend these findings to the leadership context, indicating that other-oriented perfectionism may hinder the formation of interpersonal relationships of high quality and the creation of work environments of interpersonal acceptance and trust due to the resentful and hostile reactions to errors as well as strict monitoring.

However, neither experience nor expertise can protect organizations from errors (Prümper et al., 1992). Greenleaf (1977) even describes errors as a part of human nature. Therefore, other-oriented perfectionism could be a stumbling block to organizational error management culture, which includes open communication about errors to detect and analyze them as well as learn and benefit from them (van Dyck et al., 2005). This culture has been found to be related to positive outcomes, such as organizational goal achievement and objective economic performance (van Dyck et al., 2005). However, other-oriented perfectionism among leaders may cause employees to withhold errors rather than communicate them, consequently impeding timely counteractions. By knowing these implications of other-oriented perfectionism, leaders who score high in this trait should pay special attention to being less resentful.

Similar implications apply to leaders who score high in socially prescribed perfectionism. Leaders high in either of these interpersonal dimensions of perfectionism may invest considerable efforts in monitoring mistakes. Consequently, they may lack resources concerning other responsibilities.

Overall, our results indicate that the multidimensionality of perfectionism is also reflected in its association with leadership behaviors. Whether perfectionism is detrimental or beneficial for effective leadership depends on the dimension and desired leadership behavior. Self-oriented perfectionism may be favorable in servant leadership behavior. On the contrary, socially prescribed and other-oriented perfectionistic leaders may display leadership behavior that is highly controlling.

### Theoretical Implications

Our research advances knowledge about perfectionism and leadership behavior and therefore addresses this paucity that was first mentioned 15 years ago (Flett and Hewitt, 2006). As far as we know, the present study is the first to investigate multidimensional perfectionism in leadership behavior while also considering both goal-oriented and people-centered leadership. In line with theoretical frameworks that relate leaders’ traits to their behaviors (Derue et al., 2011) and the preferred way to lead (Hogan and Kaiser, 2005), the results provide initial evidence for a perfectionism-leadership link. The findings indicate that some dimensions of perfectionism are antecedents of specific leadership behaviors, such as increased monitoring behavior (management by exception) and decreased forgiveness. This finding clearly indicates the necessity of considering the distinct relationships of the perfectionism dimensions to distinct leadership behavior.

Further, we have extended the well-established PSDM with its core assumptions concerning perfectionism and adverse social interactions to the context of leadership and leader behavior, which demonstrates the broad applicability of the model. Whereas research to date has explored perfectionism and the PSDM in relationships between people of the same level (e.g., colleagues; Kleszewski and Otto, 2020), the relationship between leaders and their followers is characterized by a hierarchical structure and thus a power difference. Accordingly, our research contributes to the perfectionism literature by exploring the link between perfectionism in a person higher in an organizational hierarchy and behavior that primarily affects people who are lower in the hierarchy and thus depend on this perfectionistic person.

### Strengths, Limitations, and Future Research

This research benefits from combining two samples and thus considers an overall sample size of 367 leaders from different occupational backgrounds or non-profit contexts; consequently, the sample can be described as heterogeneous and partially representative.

There are, nevertheless, some shortcomings that should be considered. To begin, no causal inferences can be concluded from the results because of the cross-sectional design of the study. However, although data were collected only once, we must consider that our independent variables are stable personality traits that may at least reduce the risk of having only cross-sectional data. Additionally, the hypotheses were based on well-founded theoretical models, which assume that the leader’s traits determine the performed leadership style (Hogan and Kaiser, 2005) and that they directly result in specific behaviors (Derue et al., 2011). Additionally, personality traits are conclusive predictors of various outcomes, as they develop throughout childhood and remain remarkably stable in adulthood (McCrae and Costa, 1996; Terracciano et al., 2006).

Further, future research should examine transformational leadership on the level of its facets, although it does not necessarily need to reflect them one-dimensionally. Particularly regarding the three dimensions of perfectionism, it seems likely that our assumptions of the correlations with transformational leadership behavior could have been revealed if we had examined it in additionally differentiated ways. As suggested in the discussion of the first study, relationships between transformational leadership’s subdimensions of individual consideration and idealized influence for the relational character they contain to are to be expected. Thus, future studies should apply a measure with differentiated subscales. Notably, our second study’s exploratory analyses concerning servant leadership reveal that the perfectionism dimensions are only substantially correlated with specific aspects of this multidimensional leadership behavior. An explanation for these findings could be that perfectionism can be considered as a rather narrow trait, as opposed to more global traits, such as extraversion or conscientiousness, and that leadership behavior must be measured on the same narrow level. Thus, it may be necessary for future studies to explore narrower leadership behavior, as is the case for forgiveness and humility. Accordingly, research hereafter may focus on specific leader behavior and consider constructs such as leaders’ performance feedback to delegate tasks or communicate future goals.

In our study, all data were collected by means of the self-reporting method, which may be linked with leaders’ social desirability in reporting effective leadership behavior (e.g., Atwater and Yammarino, 1992). To minimize this issue, the participants were made aware of their anonymity and instructed to respond honestly and spontaneously before they began the survey. Another problem with relying solely on self-reports is that no conclusions concerning the team members’ ratings of the presented leadership behavior can be drawn. Indeed, substantial agreement is found when self-reports are compared to observer ratings (Funder et al., 1995). In the context of work, however, the level of self-other agreement on leadership (Warr and Bourne, 1999) and leadership behaviors (Ostroff et al., 2004) is comparatively low. Thus, team members may experience their leaders as more controlling (higher in management by exception), less transformational, or less servant oriented than the leaders perceive themselves. Further research should utilize multisource and multilevel approaches, including the leaders’ and team members’ perceptions (e.g., see Hunter et al., 2013) to provide further evidence for the results of the present research. As leadership has been described as a social relationship (Stogdill, 1974), further research should focus not only on the leaders’ traits, but also on the extent to which the team members’ traits are conducive to leadership behaviors.

### Practical Implications and Concluding Remarks

Our research underscores that perfectionism in leaders is a topic worthy of further exploration. From the view of practitioners, it is of seemingly high relevance, as coaching strategies have been discussed to enable perfectionistic leaders to overcome avoidance of leadership responsibilities (Ellam-Dyson and Palmer, 2010). However, evidence-based workplace interventions are still missing. To date, the only approaches that exist are outside of the work domain. Meta-analytic evidence in clinical samples indicates that, in particular, self-oriented perfectionism can be substantially reduced in interventions, while only a medium-sized effect has been found for change in socially prescribed perfectionism (Lloyd et al., 2015). Additionally, some effects for the non-clinical population have been presented (Kearns et al., 2007).

Yet, as underscored in our research, there are positive consequences of self-oriented leaders’ perfectionism on people-centered leadership that would be highly valued in organizations; thus, calling simply for a reduction of perfectionism may fall short. Notably, a recent study has found that if employees could choose a colleague to work with, then the selected person would not be a perfectionist at all because of the potentially negative consequences on team climate (Kleszewski and Otto, 2020). Whether subordinates would also prefer non-perfectionistic leaders due to unrealistically high expectations is only speculation. Recent research at least linked leader perfectionism with leaders’ abusive behavior (Guo et al., 2020). Accordingly, Ocampo et al. (2020) demand that scholars should start undertaking empirical tests of workplace interventions to enhance the adaptive consequences of perfectionism or mitigate its maladaptive consequences.

Linking the stated findings on perfectionism to leadership exposes the importance of acquiring a deeper insight into how perfectionistic leaders shape the work environment of their subordinates and how they treat said subordinates. Based on our findings, if leaders are other-oriented or socially prescribed perfectionists, then they seem to act in a more controlling and less forgiving manner. However, the question remains: do such leaders eventually make their departments more productive and increase their organization’s success, or are they only likely to overwork their subordinates or themselves? We have provided the first evidence that various facets of perfectionism in leaders are associated with various types of leadership behavior, and we hope that these findings stimulate more research in this fruitful domain.

## Data Availability Statement

The datasets for this manuscript are not publicly available. Requests to access the datasets should be directed to KO, kathleen.otto@staff.uni-marburg.de.

## Ethics Statement

Ethical review and approval was not required for the study on human participants in accordance with the local legislation and institutional requirements. The patients/participants provided their written informed consent to participate in this study.

## Author Contributions

KO, HG, and EK developed the design of the studies. HG and EK conducted the surveys and performed the analyses. KO structured the ideas for this article and wrote the first draft. All authors read and approved the final manuscript.

## Conflict of Interest

The authors declare that the research was conducted in the absence of any commercial or financial relationships that could be construed as a potential conflict of interest.

## References

[B1] Altstötter-Gleich C. (1998). *Deutschsprachige Version der Mehrdimensionalen Perfektionismus Skala von Hewitt and Flett (1991). [German version of the Multidimensional Perfectionism Scale of Hewitt and Flett (1991)]. Unpublished manuscript, University of Koblenz-Landau, Germany.* Landau: University of Koblenz-Landau.

[B2] Atwater L. E. Yammarino F. J. (1992). Does self other agreement on leadership perceptions moderate the validity of leadership and performance predictions? *Pers. Psychol.* 45 141–164. 10.1111/j.1744-6570.1992.tb00848.x

[B3] Avolio B. J. Walumbwa F. O. Weber T. J. (2009). Leadership: current theories, research, and future directions. *Annu. Rev. Psychol.* 60 421–449. 10.1146/annurev.psych.60.110707.163621 18651820

[B4] Barbuto J. E. Fritz S. M. Matkin G. S. Marx D. B. (2007). Effects of gender, education, and age upon leaders’ use of influence tactics and full range leadership behaviors. *Sex Roles* 56 71–83. 10.1007/s11199-006-9152-6

[B5] Bass B. Riggio E. (2006). *Transformational Leadership.* Mahwah, NJ: Lawrence Erlbaum Associates.

[B6] Bass B. M. (1985b). Leadership: good, better, best. *Organ. Dyn.* 13 26–40. 10.1016/0090-2616(85)90028-2

[B7] Bass B. M. (1985a). *Leadership and Performance Beyond Expectations.* New York, NY: Free Press.

[B8] Bass B. M. (1990). From transactional to transformational leadership: learning to share the vision. *Organ. Dyn.* 18 19–31. 10.1016/0090-2616(90)90061-S

[B9] Bass B. M. Avolio B. J. (1995). *MLQ: Multifactor Leadership Questionnaire for Research: Permission Set.* Menlo Park, CA: Mind Garden.

[B10] Bono J. E. Judge T. A. (2004). Personality and transformational and transactional leadership: a meta-analysis. *J. Appl. Psychol.* 89 901–910. 10.1037/0021-9010.89.5.901 15506869

[B11] Boyatzis R. E. (1982). *The Competent Manager: A Model for Effective Performance.* New York, NY: John Wiley & Sons.

[B12] Braun S. Peus C. Weisweiler S. Frey D. (2013). Transformational leadership, job satisfaction, and team performance: a multilevel mediation model of trust. *Leadersh. Q.* 24 270–283. 10.1016/j.leaqua.2012.11.006

[B13] Britcher J. (2018). *Overcoming the Leadership Perfection Problem.* Forbes. Available online at: https://www.forbes.com/sites/forbescoachescouncil/2018/10/09/overcoming-the-leadership-perfection-problem/#591b5f3824f2 (accessed January 27, 2020).

[B14] Brown T. A. (2006). *Confirmatory Factor Analysis for Applied Research.* New York, NY: Guilford Press.

[B15] Burns D. D. (1980). The perfectionist’s script for self-defeat. *Psychol. Today* 14 34–52.

[B16] Burns J. M. (1978). *Leadership.* New York, NY: Harper and Row Publishers.

[B17] Carless S. A. Wearing A. J. Mann L. (2000). A short measure of transformational leadership. *J. Bus. Psychol.* 14 389–405. 10.1023/A:1022991115523

[B18] Clegg S. Kornberger M. Rhodes C. (2007). Business ethics as practice. *Br. J. Manag.* 18 107–122. 10.1111/j.1467-8551.2006.00493.x

[B19] Cox B. J. Enns M. W. Clara I. P. (2002). The multidimensional structure of perfectionism in clinically distressed and college student samples. *Psychol. Assess.* 14 365–373. 10.1037//1040-3590.14.3.365 12214443

[B20] De Vries R. E. (2012). Personality predictors of leadership styles and the self–other agreement problem. *Leadersh. Q.* 23 809–821. 10.1016/j.leaqua.2012.03.002

[B21] Derue D. S. Nahrgang J. D. Wellman N. Humphrey S. E. (2011). Trait and behavioral theories of leadership: an integration and meta-analytic test of their relative validity. *Pers. Psychol.* 64 7–52. 10.1111/j.1744-6570.2010.01201.x

[B22] Egan S. J. Wade T. D. Shafran R. (2011). Perfectionism as a transdiagnostic process: a clinical review. *Clin. Psychol. Rev.* 31 203–212. 10.1016/j.cpr.2010.04.009 20488598

[B23] Eisenbeiss S. A. van Knippenberg D. Boerner S. (2008). Transformational leadership and team innovation: integrating team climate principles. *J. Appl. Psychol.* 93 1438–1446. 10.1037/a0012716 19025260

[B24] Ellam-Dyson V. Palmer S. (2010). Rational coaching with perfectionistic leaders to overcome avoidance of leadership responsibilities. *Coach. Psychol.* 6 5–11.

[B25] Enns M. W. Cox B. J. (2002). “The nature and assessment of perfectionism: a critical analysis,” in *Perfectionism: Theory, Research, and Treatment*, eds Flett G. L. Hewitt P. L. (Washington, DC: American Psychological Association) 33–62. 10.1037/10458-002

[B26] Eva N. Robin M. Sendjaya S. van Dierendonck D. Liden R. C. (2019). Servant leadership: a systematic review and call for future research. *Leadersh. Q.* 30 111–132. 10.1016/j.leaqua.2018.07.004

[B27] Felfe J. (2006). Validierung einer deutschen Version des “Multifactor Leadership Questionnaire” (MLQ Form 5 x Short) von Bass und Avolio (1995). *Z. Arbeits Organisationspsychol. A O* 50 61–78. 10.1026/0932-4089.50.2.61

[B28] Ferch S. (2005). Servant-leadership, forgiveness, and social justice. *Int. J. Servant Leadersh.* 1 97–113.

[B29] Finckler P. (2017). *Transformationale Führung.* Berlin: Springer.

[B30] Fleishman E. A. (1953). The description of supervisory behavior. *J. Appl. Psychol.* 37 1–6. 10.1037/h0056314

[B31] Flett G. L. Hewitt P. L. (2002). “Perfectionism and maladjustment: an overview of theoretical, definitional, and treatment issues,” in *Perfectionism: Theory, Research, and Treatment*, eds Flett G. L. Hewitt P. L. (Washington, DC: American Psychological Association) 5–31. 10.1037/10458-001

[B32] Flett G. L. Hewitt P. L. (2006). “Perfectionism as a detrimental factor in leadership: a multidimensional analysis,” in *Inspiring leaders*, eds Burke R. J. Cooper C. L. (London: Routledge) 247–272.

[B33] Frost R. O. Marten P. Lahart C. Rosenblate R. (1990). The dimensions of perfectionism. *Cogn. Ther. Res.* 14 449–468. 10.1007/BF01172967

[B34] Funder D. C. Kolar D. C. Blackman M. C. (1995). Agreement among judges of personality: interpersonal relations, similarity, and acquaintanceship. *J. Pers. Soc. Psychol.* 69 656–672. 10.1037/0022-3514.69.4.656 7473024

[B35] Greenleaf R. K. (1970). *The Servant as Leader.* Indianapolis, IND: The Robert K. Greenleaf Center.

[B36] Greenleaf R. K. (1977). *Servant Leadership: A Journey Into the Nature of Legitimate Power and Greatness.* New York, NY: Paulist Press.

[B37] Greenleaf R. K. (1998). *The Power of Servant-Leadership.* San Francisco, CA: Berret-Koehler.

[B38] Guo L. Chiang J. T. J. Mao J. Y. Chien C. J. (2020). Abuse as a reaction of perfectionistic leaders: a moderated mediation model of leader perfectionism, perceived control, and subordinate feedback seeking on abusive supervision. *J. Occup. Organ. Psychol.* 93 790–810. 10.1111/joop.12308

[B39] Harari D. Swider B. W. Steed L. B. Breidenthal A. P. (2018). Is perfect good? A meta-analysis of perfectionism in the workplace. *J. Appl. Psychol.* 103 1121–1144. 10.1037/apl0000324 29927262

[B40] Harter S. (2002). “Authenticity,” in *Handbook of Positive Psychology*, eds Snyder C. R. Lopez S. J. (New York, NY: Oxford University Press) 382–394.

[B41] Hernandez M. (2008). Promoting stewardship behavior in organizations: a leadership model. *J. Bus. Ethics* 80 121–128. 10.1007/s10551-007-9440-2

[B42] Hewitt P. L. Flett G. L. (1991). Perfectionism in the self and social contexts: conceptualisation, assessment and association with psychopathology. *J. Pers. Soc. Psychol.* 60 456–470. 10.1037/0022-3514.60.3.456 2027080

[B43] Hewitt P. L. Flett G. L. (2004). *Multidimensional Perfectionism Scale (MPS): Technical Manual.* Toronto, ONT: Multi-Health Systems.

[B44] Hewitt P. L. Flett G. L. Mikail S. F. (1995). Perfectionism and relationship maladjustment in pain patients and their spouses. *J. Fam. Psychol.* 9 335–347. 10.1037/0893-3200.9.3.335

[B45] Hewitt P. L. Flett G. L. Mikail S. F. (2017). *Perfectionism: A Relational Approach to Conceptualization, Assessment, and Treatment.* New York, NY: Guilford.

[B46] Hewitt P. L. Flett G. L. Sherry S. B. Caelian C. (2006). “Trait perfectionism dimensions and suicide behavior,” in *Cognition and Suicide: Theory, Research, and Therapy*, ed. Ellis T. E. (Washington, DC: American Psychological Association) 215–235. 10.1037/11377-010

[B47] Hewitt P. L. Habke A. M. Lee-Baggley D. L. Sherry S. B. Flett G. L. (2008). The impact of perfectionistic self-presentation on the cognitive, affective, and physiological experience of a clinical interview. *Psychiatry* 71 93–122. 10.1521/psyc.2008.71.2.93 18573033

[B48] Hinkin T. R. Schriesheim C. A. (2008). An examination of “nonleadership”: from laissez-faire leadership to leader reward omission and punishment omission. *J. Appl. Psychol.* 93 1234–1248. 10.1037/a0012875 19025245

[B49] Hoch J. E. Bommer W. H. Dulebohn J. H. Wu D. (2018). Do ethical, authentic, and servant leadership explain variance above and beyond transformational leadership? a meta-analysis. *J. Manag.* 44 501–529. 10.1177/0149206316665461

[B50] Hogan R. Kaiser R. B. (2005). What we know about leadership. *Rev. General Psychol.* 9 169–180. 10.1037/1089-2680.9.2.169

[B51] Howell J. M. Avolio B. J. (1993). Transformational leadership, transactional leadership, locus of control, and support for innovation: key predictors of consolidated-business-unit performance. *J. Appl. Psychol.* 78 891–902. 10.1037/0021-9010.78.6.891

[B52] Hunter E. M. Neubert M. J. Perry S. J. Witt L. A. Penney L. M. Weinberger E. (2013). Servant leaders inspire servant followers: antecedents and outcomes for employees and the organization. *Leadersh. Q.* 24 316–331. 10.1016/j.leaqua.2012.12.001

[B53] Judge T. A. Piccolo R. F. (2004). Transformational and transactional leadership: a meta-analytic test of their relative validity. *J. Appl. Psychol.* 89 755–768. 10.1037/0021-9010.89.5.755 15506858

[B54] Katz D. Kahn R. L. (1952). “Some recent findings in human relations research,” in *Readings in Social Psychology*, eds Swanson E. Newcomb T. Hartley E. (New York, NY: Holt, Rinehart & Winston) 650–665.

[B55] Katz D. Maccoby N. Gurin G. Floor L. (1951). *Productivity, Supervision, and Morale Among Railroad Workers.* Oxford: Survey Research Center.

[B56] Kearns H. Forbes A. Gardiner M. (2007). A cognitive behavioural coaching intervention for the treatment of perfectionism and self-handicapping in a nonclinical population. *Behav. Chang.* 24 157–172. 10.1375/bech.24.3.157

[B57] Kleszewski E. Otto K. (2020). The perfect colleague? Multidimensional perfectionism and indicators of social disconnection in the workplace. *Pers. Individ. Dif.* 162:110016. 10.1016/j.paid.2020.110016

[B58] Kline R. B. (2005). *Principles and Practice of Structural Equation Modeling*, 2nd Edn. New York, NY: Guilford Press.

[B59] Konczak L. J. Stelly D. J. Trusty M. L. (2000). Defining and measuring empowering leader behaviors: development of an upward feedback instrument. *Educ. Psychol. Meas.* 60 301–313. 10.1177/00131640021970420

[B60] Kuhnert K. W. Lewis P. (1987). Transactional and transformational leadership: a constructive/developmental analysis. *Acad. Manag. Rev.* 12 648–657. 10.5465/AMR.1987.4306717

[B61] Lam W. Lee C. Taylor M. S. Zhao H. H. (2018). Does proactive personality matter in leadership transitions? Effects of proactive personality on new leader identification and responses to new leaders and their change agendas. *Acad. Manag. J.* 61 245–263. 10.5465/amj.2014.0503

[B62] Liden R. C. Panaccio A. Meuser J. D. Hu J. Wayne S. J. (2014). “Servant leadership: antecedents, processes, and outcomes,” in *The Oxford Handbook of Leadership and Organizations*, ed. Day D. V. (Oxford: Oxford University Press) 357–379. 10.1093/oxfordhb/9780199755615.013.018

[B63] Limburg K. Watson H. J. Hagger M. S. Egan S. J. (2017). The relationship between perfectionism and psychopathology: a meta-analysis. *J. Clin. Psychol.* 73 1301–1326. 10.1002/jclp.22435 28026869

[B64] Lloyd S. Schmidt U. Khondoker M. Tchanturia K. (2015). Can psychological interventions reduce perfectionism? A systematic review and meta-analysis. *Behav. Cogn. Psychother.* 43 705–731. 10.1017/s1352465814000162 26393777

[B65] Luthans F. (1988). Successful versus effective managers. *Acad. Manag. Exec.* 2 127–132. 10.5465/AME.1988.4275524

[B66] Mackinnon S. P. Sherry S. B. Antony M. M. Stewart S. H. Sherry D. L. Hartling N. (2012). Caught in a bad romance: perfectionism, conflict, and depression in romantic relationships. *J. Fam. Psychol.* 26 215–225. 10.1037/a0027402 22353007

[B67] Marcus D. K. Zeigler-Hill V. (2015). A big tent of dark personality traits. *Soc. Pers. Psychol. Compass* 9 434–446. 10.1111/spc3.12185

[B68] McClelland D. C. Boyatzis R. E. (1982). Leadership motive pattern and long-term success in management. *J. Appl. Psychol.* 67 737–743. 10.1037/0021-9010.67.6.737

[B69] McCrae R. R. Costa P. T. Jr. (1996). “Toward a new generation of personality theories: Theoretical contexts for the five-factor model,” in *The Five-Factor Model of Personality: Theoretical Perspectives*, ed. Wiggins J. S. (New York, NY: Guilford) 51–87.

[B70] Morris J. A. Brotheridge C. M. Urbanski J. C. (2005). Bringing humility to leadership: Antecedents and consequences of leader humility. *Hum. Relat.* 58 1323–1350. 10.1177/0018726705059929

[B71] Northouse P. G. (2016). *Leadership: Theory and Practice*, 7th Edn. Los Angeles, CA: Sage.

[B72] Nunnally J. C. Bernstein I. H. (1994). *Psychometric Theory.* New York, NY: McGraw-Hill.

[B73] Ocampo A. C. G. Wang L. Kiazad K. Restubog S. L. D. Ashkanasy N. M. (2020). The relentless pursuit of perfectionism: a review of perfectionism in the workplace and an agenda for future research. *J. Organ. Behav.* 41 144–168. 10.1002/job.2400

[B74] Ostroff C. Atwater L. E. Feinberg B. J. (2004). Understanding self-other agreement: a look at rater and ratee characteristics, context, and outcomes. *Pers. Psychol.* 57 333–375. 10.1111/j.1744-6570.2004.tb02494.x

[B75] Parolini J. Patterson K. Winston B. (2009). Distinguishing between transformational and servant leadership. *Leadersh. Organ. Dev. J.* 30 274–291. 10.1108/01437730910949544

[B76] Parris D. L. Peachey J. W. (2013). A systematic literature review of servant leadership theory in organizational contexts. *J. Bus. Ethics* 113 377–393. 10.1007/s10551-012-1322-6

[B77] Pircher Verdorfer A. Peus C. (2014). The measurement of servant leadership: validation of a german version of the servant leadership survey (SLS). *Z. Arbeits Organisationspsychol.* 58 1–16. 10.1026/0932-4089/a000133

[B78] Prümper J. Zapf D. Brodbeck F. C. Frese M. (1992). Some surprising differences between novice and expert errors in computerized office work. *Behav. Inf. Technol.* 11 319–328. 10.1080/01449299208924353

[B79] Scheel T. Otto K. Vahle-Hinz T. Holstad T. Rigotti T. (2019). A fair share of work: is fairness of task distribution a mediator between transformational leadership and follower emotional exhaustion? *Front. Psychol.* 10:2690. 10.3389/fpsyg.2019.02690 31849786 PMC6895066

[B80] Sherry S. B. Hewitt P. L. Flett G. L. Lee-Baggley D. L. Hall P. A. (2007). Trait perfectionism and perfectionistic self-presentation in personality pathology. *Pers. Individ. Dif.* 42 477–490. 10.1016/j.paid.2006.07.026

[B81] Sherry S. B. Mackinnon S. P. Gautreau C. M. (2016). “Perfectionists do not play nicely with others: expanding the social disconnection model,” in *Perfectionism, Health, and Well-Being*, eds Sirois F. M. Molnar D. S. (Cham: Springer), 225–243. 10.1007/978-3-319-18582-8_10

[B82] Smith M. M. Sherry S. B. Vidovic V. Saklofske D. H. Stoeber J. Benoit A. (2019). Perfectionism and the five-factor model of personality: a meta-analytic review. *Pers. Soc. Psychol. Rev.* 23 367–390. 10.1177/1088868318814973 30612510

[B83] Spitzer N. (2016). *Perfektionismus und Seine Vielfältigen Psychischen Folgen.* Berlin: Springer. 10.1007/978-3-662-47476-1

[B84] Stoeber J. (2014). How other-oriented perfectionism differs from self-oriented and socially prescribed perfectionism. *J. Psychopathol. Behav. Assess.* 36 329–338. 10.1007/s10862-013-9397-7

[B85] Stoeber J. (2018a). *The Psychology of Perfectionism: Theory, Research, Applications.* London: Routledge.

[B86] Stoeber J. (2018b). Comparing two short forms of the Hewitt-Flett multidimensional perfectionism Scale. *Assessment* 25 578–588. 10.1177/1073191116659740 27449052

[B87] Stoeber J. Corr P. J. Smith M. M. Saklofske D. H. (2018). “Perfectionism and personality,” in *The Psychology of Perfectionism: Theory, Research, Applications*, ed. Stoeber J. (London: Routledge) 68–88.

[B88] Stoeber J. Gaudreau P. (2017). The advantages of partialling perfectionistic strivings and perfectionistic concerns: critical issues and recommendations. *Pers. Individ. Dif.* 104 379–386. 10.1016/j.paid.2016.08.039

[B89] Stoeber J. Noland A. B. Mawenu T. W. N. Henderson T. M. Kent D. N. P. (2017). Perfectionism, social disconnection, and interpersonal hostility: not all perfectionists don’t play nicely with others. *Pers. Individ. Dif.* 119 112–117. 10.1016/j.paid.2017.07.008

[B90] Stoeber J. Otto K. (2006). Positive conceptions of perfectionism: Approaches, evidence, challenges. *Pers. Soc. Psychol. Rev.* 10 295–319. 10.1207/s15327957pspr1004_2 17201590

[B91] Stoeber J. Smith M. M. Saklofske D. H. Sherry S. B. (2021). Perfectionism and interpersonal problems revisited. *Pers. Individ. Dif.* 169:110106. 10.1016/j.paid.2020.110106

[B92] Stogdill R. M. (1974). *Handbook of Leadership: A Survey of Theory and Research.* New York, NY: Free Press.

[B93] Stone A. G. Russell R. F. Patterson K. (2004). Transformational versus servant leadership: a difference in leader focus. *Leadersh. Organ. Dev. J.* 25 349–361. 10.1108/01437730410538671

[B94] Streiner D. L. (2003). Starting at the beginning: an introduction to coefficient Alpha and internal consistency. *J. Pers. Assess.* 80 99–103. 10.1207/s15327752jpa8001_18 12584072

[B95] Sun P. Shang S. (2019). Personality traits and personal values of servant leaders. *Leadersh. Organ. Dev. J.* 40 177–192. 10.1108/LODJ-11-2018-0406

[B96] Tabachnick B. G. Fidell L. S. (2007). *Using Multivariate Statistics*, 5th Edn. Boston, MA: Pearson.

[B97] Tatoglu E. Erkutlu H. (2008). The impact of transformational leadership on organizational and leadership effectiveness. *J. Manag. Dev.* 27 708–726. 10.1108/02621710810883616

[B98] Terracciano A. Costa P. T. Jr. McCrae R. R. (2006). Personality plasticity after age 30. *Pers. Soc. Psychol. Bull.* 32 999–1009. 10.1177/0146167206288599 16861305 PMC2680603

[B99] Van Dierendonck D. (2011). Servant leadership: a review and synthesis. *J. Manag.* 37 1228–1261. 10.1177/0149206310380462

[B100] Van Dierendonck D. Nuijten I. (2011). The servant leadership survey: development and validation of a multidimensional measure. *J Bus. Psychol.* 26 249–267. 10.1007/s10869-010-9194-1 21949466 PMC3152712

[B101] van Dyck C. Frese M. Baer M. Sonnentag S. (2005). Organizational error management culture and its impact on performance: a two-study replication. *J. Appl. Psychol.* 90 1228–1240. 10.1037/0021-9010.90.6.1228 16316276

[B102] van Eeden R. (2008). Leadership styles and associated personality traits: support for the conceptualisation of transactional and transformational leadership. *S. Afr. J. Psychol.* 38 253–267. 10.1177/008124630803800201

[B103] van Knippenberg D. Sitkin S. B. (2013). A critical assessment of charismatic - transformational leadership research: back to the drawing board? *Acad. Manag. Ann.* 7 1–60. 10.1080/19416520.2013.759433

[B104] Waldman D. Bass B. Yammarino F. (1990). *The augmenting Effect of Transformational Leadership. Measures of Leadership.* West Orange, NJ: Leadership Library of America.

[B105] Warr P. Bourne A. (1999). Factors influencing two types of congruence in multirater judgments. *Hum. Perform.* 12 183–210. 10.1080/08959289909539869

[B106] Yukl G. (1999). An evaluation of conceptual weaknesses in transformational and charismatic leadership theories. *Leadersh. Q.* 10 285–305. 10.1016/S1048-9843(99)00013-2

[B107] Yukl G. (2009). Leadership and organizational learning: an evaluative essay. *Leadersh. Q.* 20 49–53.

[B108] Zaccaro S. J. Green J. P. Dubrow S. Kolze M. J. (2018). Leader individual differences, situational parameters, and leadership outcomes: a comprehensive review and integration. *Leadersh. Q.* 29 2–43. 10.1016/j.leaqua.2017.10.003

